# CD14-159C/T polymorphism in the development of delayed skin hypersensitivity to tuberculin

**DOI:** 10.1371/journal.pone.0190106

**Published:** 2017-12-27

**Authors:** Magdalena Druszczynska, Marcin Wlodarczyk, Grzegorz Kielnierowski, Michal Seweryn, Sebastian Wawrocki, Wieslawa Rudnicka

**Affiliations:** 1 Division of Cell Immunology, Department of Immunology and Infectious Biology, Institute of Microbiology, Biotechnology and Immunology, Faculty of Biology and Environmental Protection, University of Lodz, Lodz, Poland; 2 Regional Specialized Hospital of Tuberculosis, Lung Diseases and Rehabilitation, Tuszyn, Poland; 3 Center for Medical Genomics OMICRON, Jagiellonian University, Medical College, Cracow, Poland; Fundació Institut d’Investigació en Ciències de la Salut Germans Trias i Pujol, Universitat Autònoma de Barcelona, SPAIN

## Abstract

The skin tuberculin test (TST), an example of a delayed-type hypersensitivity (DTH) reaction, is based on measuring the extent of skin induration to mycobacterial tuberculin (PPD). Little is known about the genetic basis of TST reactivity, widely used for diagnosing TB infection. The study investigated the relationship of the single base change polymorphic variants in *CD14* gene (CD14(-159C/T)) with the development of DTH to PPD in BCG-vaccinated Polish Caucasian individuals. We found persistent lack of TST reactivity in about 40% of healthy subjects despite receiving more than one dose of BCG. The TST size was negatively correlated with the number of BCG inoculations. The distribution of C/T genotype was significantly more frequent among TST-negative compared with TST-positive individuals. The concentration of serum sCD14 was positively associated with mCD14 expression, but not with the TST status or CD14(-159C/T) polymorphism. A significant increase in mCD14 expression and serum sCD14 levels was found in TB group. We hypothesize that CD14(-159C/T) polymorphic variants might be one of genetic components in the response to attenuated *M*. *bovis* BCG bacilli.

## Introduction

Tuberculosis (TB) caused by *Mycobacterium tuberculosis* (*M*.*tb*) is mentioned together with AIDS and malaria among the most dangerous infectious diseases threatening human health and life. Despite the generally accepted standard of treatment TB is still a huge global epidemiological problem. Annually, there are 7–10 million new TB cases in the world, which is the cause of death of about 3 million people each year [1]. An attenuated strain of *M*. *bovis* BCG (Bacillus Calmette-Guerin) is still the only generally accepted vaccine against TB. Approximately 100 million newborns are vaccinated with BCG every year in more than 180 countries. Despite the fact that it has been more than 80 years since the first administration of BCG, the effectiveness of the vaccine is still the subject of disputes and discussions. BCG vaccination protects children from TB, especially from its most dangerous forms–miliary TB and TB meningitis but the effectiveness of the vaccine in adults does not exceed the average of 50%, ranging from 0% in India and Sub-Saharan Africa to 80% in the United Kingdom [2, 3, 4]. The WHO recommends that BCG should be given once, on the first day of life, to all children born in countries highly endemic for TB. Since 2006 BCG revaccinations of children, adolescents and adults have been discontinued as they were found ineffective and expensive [5].

A cutaneous tuberculin skin test (TST) is a classic example of a delayed-type hypersensitivity (DTH) reaction of skin to mycobacterial antigens present in PPD (purified protein derivative). The concept of the test was invented by Robert Koch in 1890 and introduced by Clemens von Pirquet in 1909 as a method for diagnosing *M*.*tb* infection [6]. Tuberculin hypersensitivity, which is a result of intensive infiltration of skin by monocytes and T and B lymphocytes is initiated by Th1 cells localized in the skin that recognize secreted proteins included in the intradermally administered PPD. Although it does not fully reflect the state of immunity to TB, it proves the development of acquired immunity to mycobacterial products, which occurs in the majority, but not all BCG vaccinated individuals. TST is currently the only assay that allows *in vivo* testing responses to mycobacterial antigens and is still considered a useful tool in TB diagnosis, however high rates of false positive reactions resulting from antigenic similarity between BCG, *M*.*tb* and environmental nontuberculous mycobacteria lower its diagnostic usefulness [6, 7, 8].

It is known that the host genetic background plays a role in the susceptibility to TB, restricting the infection or leading to active TB disease [9–12]. The genetically determined mechanisms that govern the initiation and maintenance of immune responses against *M*.*tb* can generate an imbalance between *M*.*tb* and the host immunity. The contribution of host genetic factors to the immune reactions underlying the development of DTH to tuberculin has also been widely suggested since a significant proportion of people display persistent lack of TST reactivity [4, 13–18]. One of the human genes with a possible impact on TST reactivity is the *CD14* gene encoding the CD14 receptor. CD14 molecules belong to the group of pattern recognition receptors (PRRs) recognizing the structural components of bacteria (PAMPs; pathogen associated molecular patterns) at the first steps of infection. They are expressed on the surface of macrophages, neutrophils and interstitial dendritic cells, function to aid the delivery of various ligands to TLRs, including LPS, lipoteichoic acid, ceramide, lipoarabinomannan (LAM) or poly(I:C)/double-stranded RNA [4, 19–23]. The CD14 receptors exist also in a soluble form in serum and body fluids, and appear either after the proteolytic cleavage of the membrane-anchored CD14 or are directly secreted from intracellular vesicles [24]. The initial interactions between CD14 receptors on the surface of macrophages and LAM might be a critical step in determining the outcome of infection and the development of DTH to mycobacterial antigens. The recognition of mycobacterial components by CD14 triggers a complicated series of events leading to an increased expression of proinflammatory genes that are essential for the protective immune response. The sequence of cellular immune events begins with the first exposure of the individual to tubercle bacilli. During the initial phase of the infection, antigens of the replicating mycobacteria are presented in the context of class II molecules to naive CD4^+^ T lymphocytes by infected antigen-presenting cells (APC) such as dendritic cells or monocytes. In the presence of APC-derived IL-12 and IL-18, the naive lymphocytes differentiate into T helper (Th) 1 cells, which produce IFN-γ activating macrophages and inducing a cell mediated immune response [25]. Upon the subsequent contact with the antigen, the local memory CD4^+^ and CD8^+^ T cells begin to secrete numerous cytokines responsible for the early hallmarks of inflammation [26]. About 4 hours after the antigen injection, neutrophils start to infiltrate the injection site and accumulate around the post capillary venules [27]. The influx of neutrophils decreases gradually and after 12 hours the infection site becomes infiltrated with cytokine producing T cells and macrophages. As a result of TNF-α and IFN-γ production, the endothelial cell lining undergoes remodeling and becomes permeable to plasma macromolecules. The deposited fibrin as well as the accumulation of T cells and monocytes around the injection site cause skin induration, which in humans can be detectable within 48–72 hours. T cells activated by the antigen release various lymphokines, which induce the activation and proliferation of monocytes/macrophages. Monocytes/macrophages in turn produce monokines, which regulate various cell functions and control inflammatory and immune reactions in both local and distantly located cell populations [26]. Macrophages stimulated via CD14/TLRs produce various cytokines (TNF-α, IL-1, IL-6, IL-8, IL-12, IFN-γ) and growth factors that are central to innate and acquired cellular immune responses [28].

On the basis of these findings, we focused on the relationship of single base change polymorphic variants identified in the promoter region of *CD14* gene with the development of the skin tuberculin DTH reaction in BCG-vaccinated individuals. The polymorphic site of the gene, C or T at position –159 (-159C/T), within the Sp1 transcription factor binding site, has been reported to influence membrane-bound CD14 expression on monocytes and levels of circulating soluble CD14 [29]. LeVan et al. found that the interplay between CD14 promoter affinity and the [Sp3]:[Sp1 Sp2] ratio played a critical role in regulating the transcription of the two CD14 alleles and suggested that the variation in the gene might be important for the pathogenesis of inflammatory diseases through gene-by-gene and/or gene-by-environment interactions [30].

## Materials and methods

### Study cohorts

The association of CD14 (C-159T) polymorphism with the development of DTH to PPD was assessed among 264 BCG-vaccinated HIV-negative Polish Caucasian individuals: 117 healthy, young volunteers and 147 lung disease patients, suffering from active tuberculosis (TB) or non-mycobacterial community acquired lung diseases (non-TB). All of the subjects had been vaccinated with a Brazilian *M*. *bovis* BCG strain in the past according to the Polish Government’s TB program recommendations. The study protocol was approved by the local Bioethics Committee of the Medical University of Lodz, Poland. Written consent was obtained from all individuals before study enrolment.

The group of healthy, young individuals consisted of 117 undergraduate and PhD students (aged 18–29), at the Faculty of Biology and Environmental Protection of University of Lodz, Poland, with no history of TB and no known TB contact or other immune diseases Before blood donation all the participants underwent IGRA (Interferon-gamma release assay) testing with QuantiFERON®-TB Gold In Tube test. All of the volunteers were IGRA-negative, what allowed to exclude latent *M*.*tb* infection with high probability. A summary of baseline characteristics of the studied healthy volunteers is shown in Table 1.

**Table 1:** Characteristics of the healthy young volunteer group under study.

Characteristics	Healthy young volunteers	
	Tuberculin-negative	Tuberculin-positive
	TST(-)	TST(+)
Total no. of subjects	49	68
Mean age in years (range)	23.1 (21–29)	24.2 (18–29)
Sex [no. (%) female]	37 (75.5%)	50 (73.5%)
Ethnicity	Caucasians	Caucasians
BCG inoculations [no. (%)]		
1	9 (18%)	6 (9%)
2	22 (46%)	47 (69%)
3	12 (24%)	15 (22%)
4	5 (10%)	0 (0%)
5	1 (2%)	0 (0%)
IGRA result [no. (%)]		
negative	49 (100%)	68 (100%)
positive	0 (0%)	0 (0%)

Abbreviations: BCG, Bacille Calmette-Guérin; IGRA, interferon-gamma release assay; TST, tuberculin skin test

Newly diagnosed patients with pulmonary diseases (ages, 25 to 68 years), hospitalised at the Regional Specialised Hospital of Tuberculosis, Lung Diseases and Rehabilitation in Tuszyn, Poland, were also enrolled into the study. The diagnosis was performed on the basis of the clinical presentation, a chest X-ray radiograph as well as microscopic and microbiological evaluation of sputum samples obtained by spontaneous or saline induced expectoration. The volunteers entering the study were classified as TB patients (n = 80) or non-TB patients (n = 46) on the basis of the results of triple sputum culture. Non-TB patients suffered from non-mycobacterial, community acquired pulmonary diseases and were cured with antibacterial wide-range antibiotics, whereas anti-TB drug treatment was provided to all TB patients according to the national TB programme. Demographic and clinical data collected for the hospitalized patients are shown in Table 2.

**Table 2:** Characteristics of the groups of lung disease patients under study.

Characteristics	Group of patients	
	TB	non-TB
Total no. of subjects	80	46
Mean age in years (range)	50.1 (21–68)	52.7 (21–66)
Sex [no.(%) female]	42 (52.5)	30 (65.2)
Ethnicity	Caucasians	Caucasians
BCG vaccination	80 (100)	46 (100)
Past history of TB	6 (7.5)	0 (0)
TST result [no. (%)]		
negative	32 (40)	33 (72)
positive	48 (60)	13 (28)
IGRA result [no. (%)]		
negative	31 (39)	40 (87)
positive	48 (60)	6 (13)
indeterminate	1 (1)	0 (0)

Abbreviations: BCG, Bacille Calmette-Guérin; IGRA, interferon-gamma release assay; TST, tuberculin skin test

### Tuberculin skin testing

After the collection of blood for DNA extraction and IGRA testing, all individuals were screened with 2 tuberculin units (TU) of *M*.*tb* PPD RT-23 (Statens Serum Institute, Copenhagen, Denmark), injected intradermally in the forearms by trained medical staff as described previously [7, 8]. The reaction was considered positive if the diameter of skin induration measured 72 hours after PPD administration was equal or greater than 10 mm.

### QuantiFERON®-TB Gold In Tube assay

Just before the screening with tuberculin, the interferon-gamma release assay (IGRA) was performed in all individuals. The QuantiFERON®-TB Gold In Tube (Cellestis Limited, Carnegie, Australia) was conducted according to manufacturer’s instructions as described in detail previously [7, 8]. The result was considered positive if the difference between IFN-γ level in plasma incubated with TB antigen and Nil control was both ≥ 0.35 IU/ml and ≥25% of Nil control value.

### Genomic DNA extraction

Genomic DNA was isolated from ethylenediaminetetraacetic acid (EDTA)-anticoagulated blood samples using a QIAamp^®^ DNA Blood Mini Kit (Qiagen^®^, Hilden, Germany) according to the instructions of the manufacturer. After extraction, DNA was quantified by spectrophotometry, checked for purity and stored at –20°C until further analysis.

### CD14(-159C/T) genotyping

The DNA samples of 264 subjects were genotyped for CD14 (-159C/T, rs2569190), polymorphism using the following 4 primers: for C allele, a forward primer cfors: 5^’^-CTC CAG AAT CCT TCC TGT TAC GAC-3^’^ and a reverse primer cdp2: 5’-TTG GTG CCA ACA GAT CAG GTT CAC-3’, for T allele, a forward primer cdp1: 5’-TTG GTG CCA ACA GAT CAG GTT CAC-3’ and a reverse primer trevs: 5’-TGT AGG ATG TTT CAG GGA GGG GTA-3’. The primers cfors and trevs were designed from the published sequence so that an additional mismatch was inserted at the penultimate 3^’^ nucleotide to increase the specificity of the amplification reaction [31]. The C(-159)T CD14 polymorphism determined with an allele specific PCR method was performed in a total volume of 10 μl (50–100 ng of genomic DNA, 1 X PCR buffer with 1.5 mM MgCl_2_, 200 μM of nucleotides, 0.5 U Taq polymerase and 0.5 μM primers). After an initial denaturation at 95°C for 5 min, 30 cycles were run at 95°C for 30 s and 72°C for 1 min, and thereafter a final extension at 72°C for 5 min. Amplified products were visualized by electrophoresis in 2% agarose gel stained with ethidium bromide (10 mg/ml). The assay yielded a 381-bp band for the T allele and a 227-bp band for the C allele. Product bands were visualized on a Gel Doc 2000 gel documentation system (Bio-Rad, Hercules, CA, USA).

### Serum sCD14 levels

The concentration of the soluble CD14 receptor in serum was analyzed by the immunoenzymatic method (ELISA) according to the producer^’^s (R&D Systems, Minneapolis, MN, USA) manual for the Human sCD14 Kit. The readouts were made at 450 nm using a multifunctional counter Victor 2 (Wallac Oy, Turku, Finland).

### Monocyte mCD14 expression analysis

Samples of heparinized blood were used for flow-cytometry analysis of monocyte mCD14 expression as described previously [23]. Briefly, the peripheral blood mononuclear leukocytes (10^5^) were incubated (30 min, 4°C) with monoclonal mouse FITC-conjugated IgG2a anti-human CD14 antibody (BD Biosciences, USA). A total of 10000 cells were analyzed using a FACScan (BD) and Flow Jo software 7.2.2. (Tree Star Inc., USA). Isotype control antibodies were used as a control for non-specific binding of antibodies. The mCD14 density was expressed as mean fluorescence intensity (MFI) of anti-CD14 treated samples diminished by MFI of isotype matched negative control.

### Statistical analysis

Statistical analysis was performed using the R statistical software. All generalized linear models (both linear and logistic) were fitted and analyzed using the 'glm' function in package 'stats'. The presented p-values correspond to Wald's test of significance of the coefficient in the linear model. Presented correlations are Pearson product moment coefficients unless otherwise stated. Where appropriate, the significance of correlations was further confirmed using both the Spearman's and Kendall's rank based methods. Hardy-Weinberg equilibrium was analyzed using package 'HardyWeinberg'. Equality of proportions of TST(+) and TST(-) individuals with different genotypes was tested using function 'prop.test'.

## Results and discussion

### Characteristics of study participants

**Fig 1:**
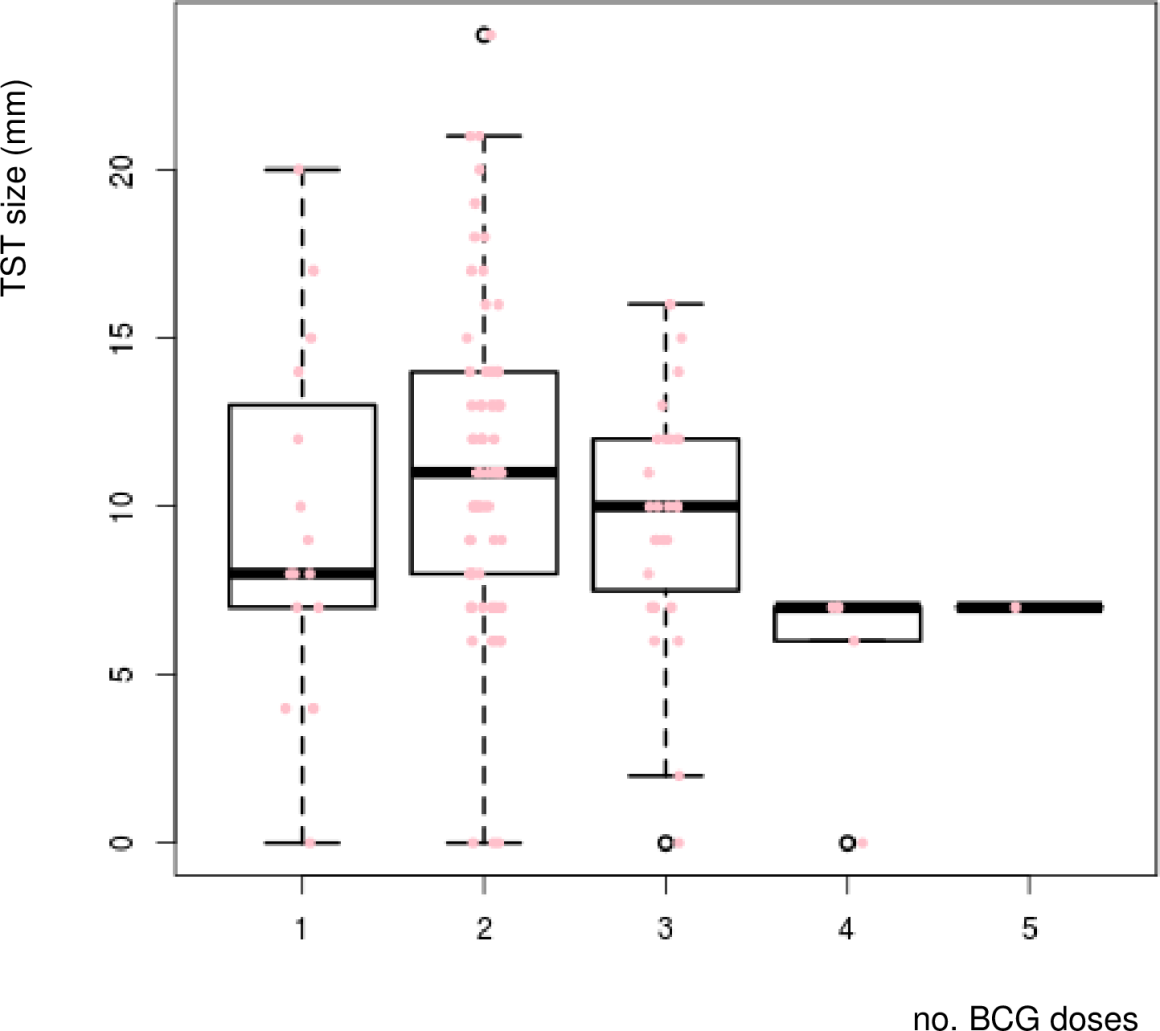
Correlation between TST size and the number of BCG doses in the group of healthy, young individuals.

The baseline characteristics of the studied groups are shown in Tables 1 and 2. A total of 117 healthy, young IGRA-negative volunteers of Polish Caucasian ethnic group consented to take part in the study (Table 1). Mean age of the volunteers was 23.7 years (SD, 1.8 years) and the sex ratio was 3:1 (F:M). The individuals were defined either as tuberculin-negative (TST(-)) or tuberculin-positive (TST(+)) on the basis of the diameter of skin induration to intradermally administered PPD. The reactivity ranged from 0 to 24 mm with a median size of 10 mm. Of 117 individuals, 68 (58%) responded to tuberculin with skin induration of more than 10 mm and were defined to be TST(+). The majority of studied individuals had received two or three doses of BCG vaccine in their life. In the TST(-) group, 9 (18%) participants had received BCG vaccine only once at birth, 22 (46%) individuals had received a second BCG dose at the age of 6, and 12 (24%), 5(10%) and 1 (2%) volunteers had been additionally revaccinated with BCG three, four and five times, respectively (Table 1). Among 68 TST(+) participants, 6 (9%), 47 (69%) and 15 (22%) subjects had received one, two or three BCG doses, respectively. The number of BCG inoculations was negatively associated with the size of the reaction to PPD at a borderline statistical significance level (p = 0.06), which corresponds to the test based on the estimate of the product-moment (Pearson’s) correlation coefficient (Fig 1).

Further analysis of the distribution of TST sizes according to the number of BCG inoculations showed that individuals with skin induration diameters of 10–14 mm or above 15 mm had received in the past fewer doses of the vaccine than those with smaller TST sizes (Table 3).

**Table 3:** Distribution of TST sizes according to the BCG inoculations.

BCG doses (n)	Healthy young individuals			
	n (%)			
	TST size range (mm)			
	negative		positive	
	≤4	5–9	10–14	≥15
1	3 (33)	6 (15)	3 (6)	3 (18)
2	3 (33)	19 (48)	35 (69)	12 (71)
3	2 (22)	10 (25)	13 (25)	2 (11)
4	1 (12)	4 (10)	0 (0)	0 (0)
5	0 (0)	1 (2)	0 (0)	0 (0)

Abbreviations: BCG, Bacille Calmette-Guérin; TST, tuberculin skin test

Among 17 young, healthy individuals with the largest TST diameters, 3 (18%) volunteers had been vaccinated with BCG once, 12 (71%) twice, and 2 (11%) three times. A similar tendency was noted in the group of volunteers with TST sizes of 10–14 mm, among whom the percentage of individuals vaccinated with one, two or three BCG doses was 6% (3/51), 69% (35/51) and 25% (13/51), respectively. None of the volunteers with positive TST results had received more than three BCG inoculations. On the contrary, among TST(-) individuals having a diameter of skin induration of between 5–9 mm or less than 4 mm, 13% (5/40) or 12% (1/9) volunteers had been vaccinated with BCG four or five times (Table 3). As noted in Table 1, all healthy young volunteers showed negative IGRA test that measure IFN-γ released from lymphocytes exposed to specific *M*.*tb* antigens.

The main demographic characteristics of patients with pulmonary diseases, suffering from active TB (TB) or non-mycobacterial community acquired lung diseases (non-TB) included in the study are shown in Table 2. The mean age of the patients in each group was similar, at 50.1±17.6 and 52.7±17.3 years, respectively. Six out of 80 (8%) TB patients had a past history of healed pulmonary TB. The TST sizes in the TB patient group ranged from 0 to 35 mm with a median of 10,5 mm and from 0 to 25 mm (median of 0 mm) among non-TB patients. Positive reactions to PPD with a diameter of skin induration greater than 10 mm were developed significantly more frequently by TB patients (60%) than among non-TB patients (28%) (p = 0.0006; one-sided proportion test) (Table 2). The frequency of IGRA positives was 60% among TB patients and 13% among non-TB patients (p = 0.00001). One out of 80 (1%) TB patients had an indeterminate result of an IGRA test (Table 2). Among 48 TST(+) patients with TB 37 had positive IGRA results (IGRA(+)), and among 13 TST(+) patients from non-TB group 4 were IGRA(+).

**Fig 2:**
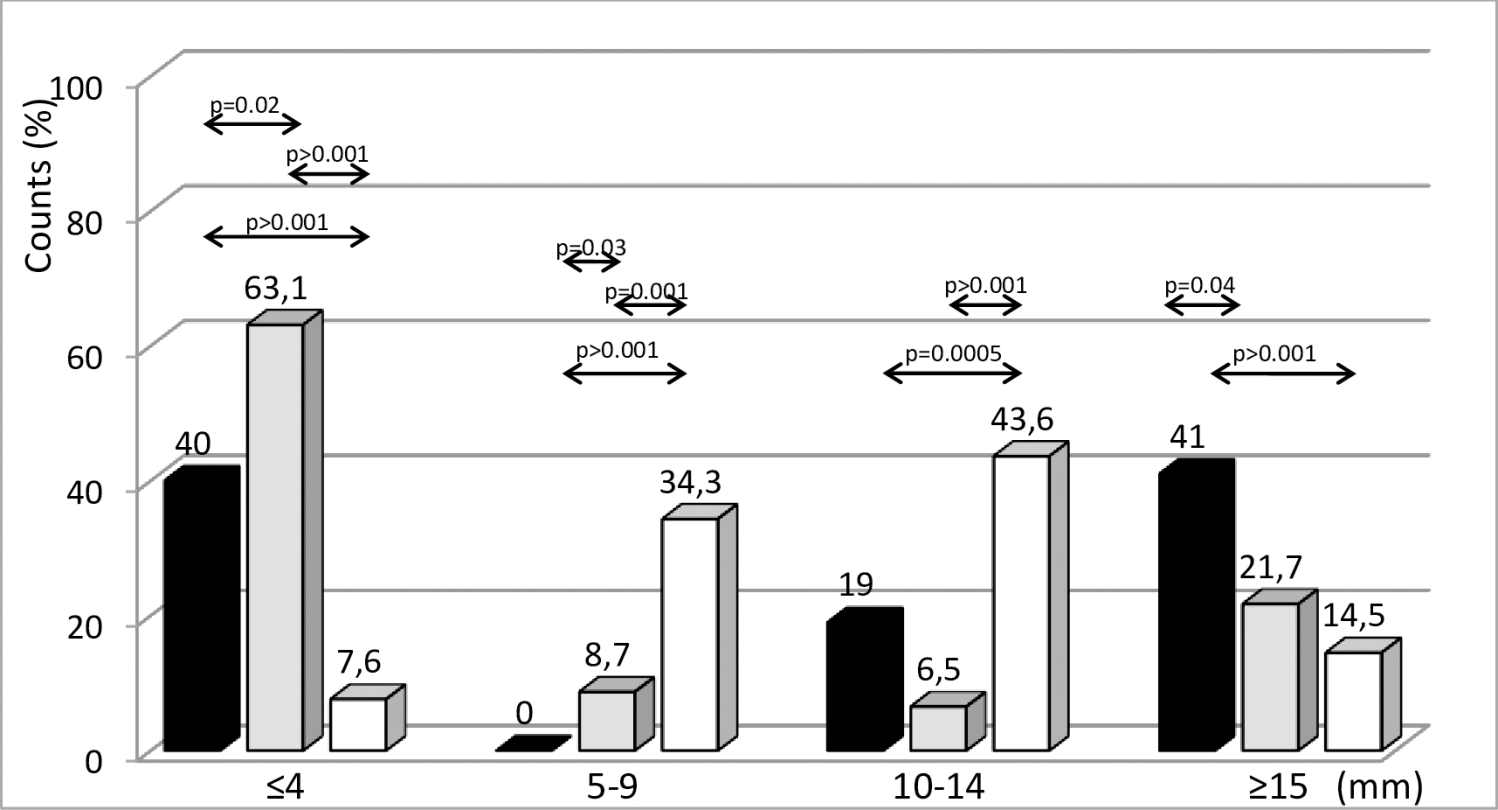
Distribution of TST sizes among the study groups: TB patients (black bars), non-TB patients (grey bars) and young healthy volunteers (white bars).

Analysis of the TST in four size ranges (≤ 4 mm, 5–9 mm, 10–14 mm, ≥ 15 mm) including data obtained for all volunteers participating in the study showed a great variation in the distribution of TST sizes in the studied groups (Fig 2). The distribution of TST results was not identical in any of the studied groups (Fig 2) as estimated with Kolmogorov-Smirnov test. The percentage of TST sizes among healthy young volunteers was significantly different from the distribution observed either in TB or non-TB patients in each studied category. Compared to TB patients, the percentage of TST sizes above 15 mm decreased to 21.7% in non-TB patients and 14.5% in healthy, young volunteers.

### CD14(-159C/T) genotypes in TST(-) and TST(+) individuals

The distribution of CD14-159C/T genotypes in the TST(-) and TST(+) individuals from the studied groups is shown in Table 4. There was no evidence to reject the Hardy-Weinberg equilibrium (HWE) hypothesis in both TST(-) and TST(+) groups at the standard significance level of 0.05.

**Table 4:** Frequency of CD14(-159C/T) genotypes in TST(-) and TST(+) individuals from the studied groups.

Group of study	CD14	Total	TST result		p	OR (95% CI)
			n (%)			
	(-159C/T) genotype	n (%)	-	+		
Healthy volunteers	C/C	46 (39)	14 (29)	32 (47)	0.04	0.4 (0.169;0.830)
	C/T	58 (50)	30 (61)	28 (41)	0.03	2.3 (1.064;4,778)
	T/T	13 (11)	5 (10)	8 (12)	0.79	0.9 (0.261;2.782)
	P^HWE^	0.404	2.716	0.778		
TB patients	C/C	29 (36)	10 (31)	19 (40)	0.44	0.7 (0.269;1.785)
	C/T	32 (40)	15 (47)	17 (35)	0.31	1.6 (0.646;4.005)
	T/T	19 (24)	7 (22)	12 (25)	0.74	0.8 (0.291;2.431)
	p^HWE^	0.1	0.759	0.06		
Non-TB patients	C/C	15 (33)	10 (30)	5 (38)	0.85	0.8 (0.208;2.934)
	C/T	24 (52)	20 (60)	4 (31)	0.13	3.5 (0.880;13.612)
	T/T	7 (15)	3 (10)	4 (31)	0.16	0.2 (0.042;1.197)
	p^HWE^	0.606	0.121	0.169		

The heterozygous C/T CD14(-159C/T) genotype was found more frequently among TST(-) compared with TST(+) individuals in all groups under study, however a statistically significant difference was noticed only in the group of healthy young individuals (Table 4). In this group, the C/T heterozygosity occurred significantly more frequently in healthy volunteers with negative skin reactions to PPD (61%) than TST(+) individuals (41%) (p = 0.03). The C/C CD14(-159C/T) genotype significantly was more frequent among TST(+) compared with TST(-) individuals, at 29% vs 47% (p = 0.04). In the TB patient and non-TB patient group no significant differences were found. The frequency of the T/T genotype was low in both TST(-) and TST(+) individuals (Table 4). In the study the distribution of CD14(-159C/T) genotypes were analyzed among IGRA(-) and IGRA(+) individuals from TB and non-TB group (Table 5). There was no difference in the frequency of the genotypes between IGRA(+) and IGRA(-) patients with active TB or non-mycobacterial lung diseases.

**Table 5:** Frequency of CD14(-159C/T) genotypes in IGRA(-) and IGRA(+) individuals from TB and non-TB groups.

Group of study	CD14	Total	IGRA result		p	OR (95% CI)
			n (%)			
	(-159C/T) genotype	n (%)	-	+		
TB patients	C/C	28 (36)	12 (38)	16 (33)	0.62	1.3 (0.493;3.231)
	C/T	32 (40)	12 (38)	20 (42)	0.79	0.9 (0.351;2.225)
	T/T	19 (24)	7 (24)	12 (25)	0.64	0.9 (0.301;2.540)
	p^HWE^	0.1	0.25	0.26		
Non-TB patients	C/C	15 (33)	12 (30)	3 (50)	0.33	0.4 (0.075;2.435)
	C/T	24 (52)	22 (55)	2 (33)	0.32	2.4 (0.400;14.908)
	T/T	7 (15)	6 (6)	1 (17)	0.92	0.9 (0.087;8.941
	pHWE	0.606	0.42	0.54		

### Association analysis of CD14(-159C/T) polymorphism with DTH to PPD using logistic regression

In the group of healthy individuals we considered a logistic model with the TST status as the dependent variable and two covariates: the CD14(-159C/T) genotype and the number of BCG doses. Where possible, we tested the effects of the mutation in codominant, dominant, recessive and additive models (Table 6).

**Table 6:** Association analysis of the CD14(-159C/T) polymorphism with DTH to PPD (adjusted by BCG doses) in healthy, young volunteers using logistic regression.

Group of study	Model			
	effect size (p-value)			
	Co-dominant; AIC = 160.89	Dominant; AIC = 159.47	Recessive; AIC = 163.15	Additive; AIC = 161.38
Healthy volunteers	C/T -0.8498 (0.0419)	T/T -0.7619 (0.0578)	T/T 0.1101 (0.8566)	-0.3909 (0.1818)
	T/T -0.3718 (0.5715)			

Our analysis showed that the mutated T allele had a negative effect on the reactivity to tuberculin in the dominant model (p = 0.057; effect size = -0.7619), and that the C/T genotype also had a significant negative effect in the co-dominant model (p = 0.041; effect size = -0.8498) (Table 6). The dominant model, which had the lowest AIC value (159.47) was accepted as best explaining the TST status. The results demonstrated that a single copy of the T allele was enough to associate with skin reactivity to PPD. Having at least one mutant allele for the CD14(-159C/T) polymorphism significantly decreased the DTH development.

### Monocyte mCD14 expression and serum sCD14 levels in TST(-) and TST(+) individuals

A statistical analysis of the data showed a significant increase in the mCD14 expression on monocytes in TB patients (10859±3054 MFI) as compared to non-TB patients (9025±2684 MFI) (p = 0.0007) (Table 7).

**Table 7:** Monocyte mCD14 expression and serum sCD14 levels in TST(-) and TST(+) individuals.

Study group	mCD14			sCD14		
	(MFI)			(ng/ml)		
	Total	TST result		Total	TST result	
		-	+		-	+
Healthy volunteers	n.d.	n.d	n.d.	1460±455	1452±416	1546±484
TB patients	10859±3054	11568±3625	10366±2511	2198±371	2240±330	2170±396
Non-TB patients	9025±2684	8570±2809	10146±2030	2024±398	2100±346	1935±399

n.d.—not determined

However, the level of sCD14 was significantly higher in sera from both TB (2198±371 ng/ml) and non-TB patients (2024±398 ng/ml) compared to healthy volunteers (1460±455 ng/ml; p = 6.822e-14, p = < 2.2e-16). There were no significant differences in the mCD14 expression on monocytes from TST(-) and TST(+) individuals from both TB and non-TB groups. Similarly, the average concentration of sCD14 in sera from TST(-) volunteers was equal to that observed in TST(+) subjects in each group under study (Table 7).

**Fig 3:**
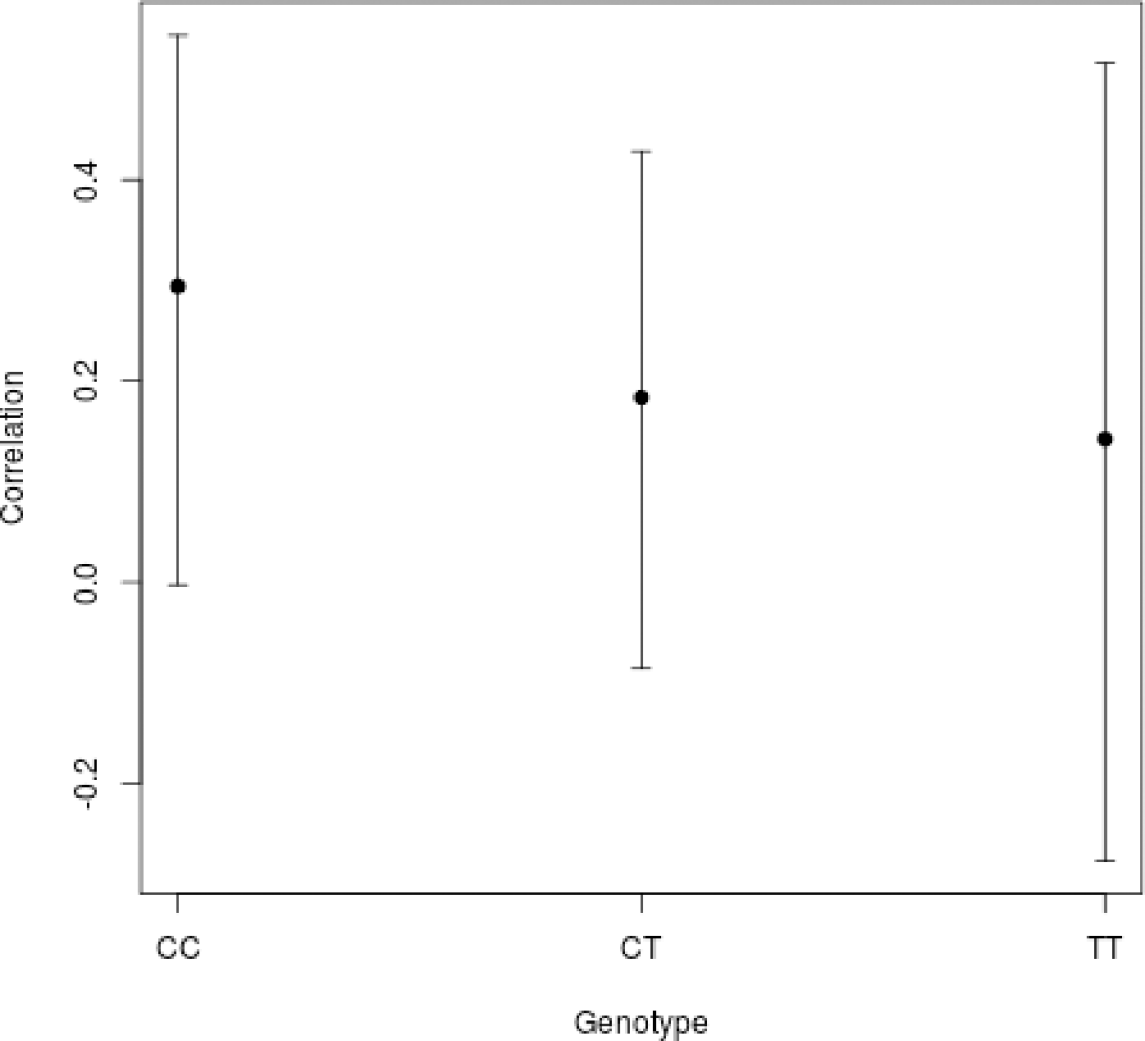
Correlation between serum sCD14 levels and mCD14 expression in individuals with C/C, C/T and T/T CD14(-159C/T) genotypes.

We found that the serum sCD14 concentration was positively associated with monocyte mCD14 expression (Spearman's r = 0.19, p = 0.02) in TB and non-TB patients. The association analysis of mCD14 and sCD14 levels with the CD14(-159C/T) polymorphism demonstrated that a correlation was observed solely among the C/C homozygotes (p = 0.05; Spearman's r = 0.28), but not among the C/T (p = 0.19; Spearman's r = 0.17) or T/T carriers (p = 0.51; Spearman's r = 0.13) (Fig 3). However, observed correlation is very weak and should be carefully interpreted.

### Association analysis of CD14(-159C/T) polymorphism with sCD14 and mCD14 levels using linear regression

We have evaluated the effect of the CD14 genotype on the serum sCD14 level and monocyte mCD14 expression using Gaussian regression with sCD14 and mCD14 levels as the dependent variable and the CD14 genotype as the covariate, and three different inheritance models (dominant, recessive and co-dominant). In the cohort of healthy volunteers we observed a significant negative effect of the T/T genotype in the recessive model (effect size = -275, p = 0.04) (Table 8).

**Table 8:** Association analysis of sCD14 levels or mCD14 expression with the CD14(-159C/T) genotype using linear regression.

	sCD14			CD14 regression model	
	(ng/ml)			effect size	
Study group	CD14 genotype			(p-value)	
	C/C	C/T	T/T	dominant	recessive
Healthy volunteers	1472±366	1506±491	1216±529	-19.17	-274.49
				(0.825)	(0.04)
TB patients	2173±327	2185±347	2257±475	39.05	78.19
				(0.654)	(0.426)
Non-TB patients	1983±513	2079±284	2117±233	104.81	74.65
				(0.367)	(0.624)
	mCD14			CD14 model	
	(MFI)			effect size	
Study group	CD14 genotype			(p-value)	
	C/C	C/T	T/T	dominant	recessive
TB patients	11294±3349	10657±2489	10498±3565	-692.3	-462.4
				(0.337)	(0.584)
Non-TB patients	9016±2333	9061±2800	8930±3372	14.53	-112.6
				(0.987)	(0.92)

Since we could not replicate our finding as far as the effect of the CD14 variant on the sCD14 concentration in the cohort of healthy volunteers, we have further evaluated the effect of the CD14(-159C/T) polymorphism on both sCD14 and mCD14 concentration in the TB vs. non-TB cohort. To this aim, we first regressed out the effect of mCD14 on sCD14 by applying a standard linear model. Furthermore, we noted that the distribution of residuals is dependent on the CD14 genotype–namely, for homozygous minor there is no evidence to reject the hypotheses that residuals follow a normal distribution, whereas for the homozygous minor we reject the null hypothesis on normality of the residuals. Due to this fact, we used both the Mood's median test and Kruskal-Wallis test to compare the medians in the three groups: homozygous major (median = 72.34), heterozygous (median = 45.23) and homozygous minor (median = 194.00), but we were unable to reject the null due to the lack of power. Nevertheless, we were able to test the recessive effect of the CD14 variant using Wilcoxon test–that is we were able to reject the null in three cases–homozygous major vs homozygous minor (p = 0.04), heterozygous vs homozygous minor (p = 0.04), homozygous major and heterozygous vs homozygous minor (p = 0.02). In what follows, we aimed to seek for the source of this effect of the CD14(-159C/T) polymorphism on the residuals (but not on serum CD14 nor monocyte CD14 levels). We used Genotype Tissue Expression data through http://www.gtexportal.org to test the impact of rs2569190 on CD14 mRNA expression. In five tissues, where CD14 is well enough expressed (liver, lung, spleen, adrenal gland and whole blood) rs2569190 is a significant eQTL for CD14 only in liver (p = 0.024). This supports our hypothesis that the observed effect might be hepatocyte-specific and thus detectable in the healthy cohort in serum CD14 but masked by monocyte/macrophage activity in the TB vs non-TB cohort.

## Discussion

Although the cellular components involved in the DTH reaction have been well described, the molecular mechanisms responsible for the protection provided by the BCG vaccine have not been fully understood. The accurate understanding of highly complex mechanisms of specific immunity to *M*.*tb* infection including defensive reactions and processes involved in TB pathology is necessary to develop new ways of preventing TB and vaccines which would be more effective than currently used one containing attenuated *M*. *bovis* BCG bacilli. It is also necessary to find effective tools for the rapid assessment of protective properties of the vaccine which is already used and those currently being developed, the effectiveness of which will be evaluated no sooner than after several years.

Immune parameters responsible for varying protective effectiveness of BCG vaccine remain unexplained. The heterogeneity has been ascribed to several factors such as: biological variability of BCG vaccine strains, route of vaccine administration, dose used, exposure to environmental mycobacteria and host-related factors including nutritional status, infections as well as genetic background [13, 14, 32]. There are differences about the interpretation of the TST results in different populations [33]. In Poland, the country with moderate TB prevalence and obligatory BCG immunization, the size of induration of at least 10 mm is considered positive [8, 34]. A negative TST does not actually mean susceptibility to *M*.*tb* infection or TB disease. On the contrary, the risk of developing active TB for individuals with sustained *M*.*tb* exposure, who displayed persistent lack of skin DTH reactivity, was shown to be extremely small [35]. Moreover, a significant percentage of the individuals remained uninfected most likely due to the resistant immune status that underlined the effective initiation and maintenance of immune response against *M*.*tb* [16].

There is accumulate evidence that human genetics plays a key role in determining individual potency in developing an effective innate and adaptive immune responses during M.tb infection and consequently conditioning susceptibility to TB disease. The interindividual variability is noticeable at the early stage of infection, as approximately 20–30% of subjects exposed to *M*.*tb* do not become infected [16, 36]. Moreover, about one-third of the global population is infected with *M*.*tb*, but only an estimated 10% of the infected develop clinical TB during their lifetime. Our results suggest the involvement of the host genetic component in the immune reactions induced by mycobacteria. We confirmed our previous results [4] that about 40% of healthy young *M*. *bovis* BCG-vaccinated individuals did not develop delayed type hypersensitivity to injected PPD, although approximately 80% of them had received more than one dose of the BCG vaccine in the past. In this group, the number of BCG inoculations was negatively correlated with the size of the reaction to PPD at a borderline statistical significance level. Young individuals with positive tuberculin reactions differed from tuberculin-negative subjects in the distribution of CD14(-159C/T) genotypes. The C/C genotype was more prevalent among the subjects with skin hypersensitivity to PPD, while the C/T genotype dominated among tuberculin-negative volunteers. The logistic regression analysis showed that in the group of young healthy blood donors the mutated T allele had a negative effect on the reactivity to tuberculin in a dominant model, and that the C/T genotype had a significant negative effect in a co-dominant model. Such association was not observed in the group of patients with pulmonary diseases, suffering from active TB or non-mycobacterial community acquired lung diseases. This incompatibility could be explained by the older age of patients with lung diseases, compared to young healthy volunteers. The suggestion could be proposed on the basis of the results of our previous research showing that the percentage of positive TST decreased significantly with the age of TB patients [8]. The probability of such an explanation weakens the lack of such trend in patients with non-mycobacterial lung diseases. Interestingly, there is a similar percentage of TST negative in healthy, young group and TB patients. The statistical analysis (based both on a generalized linear model and a linear mixed model) performed in the whole group under study considering the CD14(-159C/T) genotype and the age as covariates confirmed a significant negative effect of the T allele on the skin reactivity to PPD in a dominant model (p = 0.0316, effect size = -0.60621) as well as a negative effect of the C/T genotype in a co-dominant model (p = 0.00796, effect size = -0.79416).

Although the biological effect of the CD14(-159C/T) polymorphism remains unclear, the SNP has been associated with several diseases such as TB, brucellosis, chronic peridonitis, chronic chlamydial infection and Crohn disease [37–40]. There are also several reports on the association of the CD14 gene variants with the prevention or severity of atopy [41]. Various studies implicated both the T and C CD14 alleles as risk factors, while others found no such association [42–44]. In our study, the logistic regression analysis demonstrated that a single copy of the mutated T allele was enough to decrease the possibility of DTH development, however, our results do not allow us to know if there is a link between the CD14 (-159C/T) polymorphism and TB susceptibility. The study by Rosas-Taraco et al. showed a higher frequency of T/T CD14 (-159C/T) genotypes in patients with pulmonary TB than in healthy control subjects [45]. In turn, other researchers showed an increase in TB risk in carriers of T/T homozygote variant among Asians, but not among Caucasians [46].

The CD14(-159C/T) polymorphism (also referred to as to -260C/T) located at the promoter region of the *CD14* gene has been found to be a functional genetic variant, albeit its effect on expression of the mRNA appears to be highly tissue specific (as supported by the GTEx eQTL data). LeVan et al. reported that the mutated T allele had a decreased affinity for DNA/protein interactions at a GC box containing a binding site for SP1, SP2, and SP3 transcription factors, thereby the homozygous T/T genotype diminished the affinity of the nuclear factors binding to the CD14 promoter and enhanced the transcriptional activity of the CD14 gene [30]. Similarly, Kang et al found that the promoter activity of the T allele was higher than that of the C allele in transfected K562 and BEAS-2B cells [47]. Consistently, Härtel et al. showed that the CD14(-159C/T) polymorphism was associated with soluble CD14 expression, which might influence the balance of pro- and anti-inflammatory immune responses in healthy term neonates [48]. After *in vitro* stimulation of cord blood cultures with lipopolysaccharide, the carriers of the T allele had higher levels of sCD14 and increased concentrations of IL-6 compared with the C allele carriers. In our study, sCD14 or mCD14 levels did not differ between the T/T and C/C genotypes either among healthy blood donors, TB or non-TB patients, however we confirmed our previous study and found significantly higher levels of the proteins among patients with TB [23]. Our data are consistent with results by others [45, 49, 50]. It is very likely that the overexpression of macrophage mCD14 may be significant in the TB development. As it has been shown, *M*.*tb* bacilli grow more rapidly in macrophages presenting a high density of CD14 molecules than in CD14-low cells [51]. On the other hand, the increase in sCD14 and mCD14 levels may be a part of *M*.*tb*-driven inflammatory response of monocytes and macrophages. Serum sCD14 levels were found elevated also in patients with brucellosis, leishmaniasis, HCV or HBV infection [52–55]. Although leukocytes represent the major source of sCD14, it is also produced by hepatocytes, which are the main source of many acute-phase proteins [56]. It cannot also be excluded that other SNPs identified within the *CD14* promoter region (1619G/A, -1359T/G, -1145A/G) might affect *CD14* gene expression and contribute to differences in the levels of encoded proteins. Moreover, longitudinal studies have suggested that an association between CD14 polymorphisms and sCD14 levels might be age-dependent since a relation of CD14 genotypes with sCD14 was found up to 1 year of age, but not at birth [57].

The exact biological function of soluble CD14 is so far unknown. As an acute-phase protein, sCD14 acts as a negative regulator of human T cell activation and function. It was found *in vitro* that elevated sCD14 levels inhibited the binding of ligands to mCD14 and hence blocked cellular activation [58]. It was demonstrated that elevated sCD14 levels could inhibit *M*.*tb* internalization or interaction of the mycobacterial component with the membranous CD14 form through competitive inhibition [59, 60]. The soluble CD14 form impaired antigen-mediated proliferation of PBMCs and anti-CD3-mediated proliferation of CD4^+^CD8^-^, CD4^-^CD8^+^ and CD4^+^CD8^+^ T cells, which was a consequence of a marked inhibition of IL-2 production [58, 61]. It was also found to diminish secretion of IFN-gamma and induce a progressive accumulation of the inhibitory protein IκB-α, which was responsible for the cytoplasmic retention of NF-κB in an inactive form in unstimulated cells [58]. This observation is in accordance with the results by Kang et al. showing significantly lower levels of IFN-γ produced by PPD stimulated PBMCs of healthy individuals with T/T than those with C/C CD14 (-159C/T) genotypes [47]. Taking into account that IFN-γ is thought to be a principal mediator of macrophage activation and resistance to intracellular *M*.*tb* bacilli, it is likely that the impairment in IFN-γ production might have a negative impact on the development of DTH to PPD [62]. One of our previous studies conducted in BCG-vaccinated healthy volunteers as well as the reports of other authors revealed that circulating lymphocytes from TST-positive subjects produced significantly more IFN-γ but less IL-10 in response to PPD than the cells from TST-negative individuals suggesting that the development of DTH to PPD may depend on an immanent tendency of polarization Th1/Th2 response to mycobacterial antigens [63]. This prompted us to ask whether there is a link between the CD14(-159C/T) polymorphism and IFN-γ production in response to specific *M*.*tb* antigens evaluated in the IGRA test. The analysis could be performed for TB and non-TB patients, among whom, 60% and 13% of positive IGRA results, respectively, were found. However, there was no relationship between the IGRA result and the polymorphism studied. This result is not surprising because the CD14 molecule is a marker of monocytes/macrophages, whereas the IFN-γ, measured in the IGRA test, is mainly produced by the sensitized CD4^+^ T helper lymphocytes.

The activation and recruitment of monocytes into the area of the inflammatory tuberculin reaction is a crucial step in the development of DTH, which is an excellent model of integrated innate and adaptive immune responses to *M*.*tb*. Recognition of mycobacterial antigens by CD14 receptors, in both membranous and soluble forms, is a key element of the first line of defense and an important link to the specific phase of the adaptive immunity. The initiation of the signal cascade leads to the release of monocyte inflammatory cytokines responsible for effective elimination of the pathogen. Surface CD14 expression seems to be a factor that regulates the growth of *M*.*tb* in subpopulations of human macrophages. Taking this issue into account in our previous study [4] we set up the original hypothesis concerning the relationship between the expression of monocyte signal transduction receptors and cellular immunity to the BCG vaccine assessed on the basis of delayed type hypersensitivity (DTH) to tuberculin. A group of healthy young people who were subjected to compulsory BCG vaccination in infancy and school age and who had never suffered from TB was selected as a model for this study. The development of specific cellular immunity was measured on the basis of the delayed type hypersensitivity to PPD before blood donation. We found that young people, aged 18–30, undergoing antituberculosis BCG vaccination according to the vaccination schedule in Poland, vary in their response to tuberculin. Only slightly more than 60% of them exhibit a positive reaction to PPD, while the others remain tuberculin-negative. Diversified response of TST(+) and TST(-) volunteers to subcutaneous injection of PPD was accompanied by a slight difference in the expression of the membrane receptor mCD14 on monocyte fractions prepared on the basis of the adherent properties of these cells, which contained an average 79% CD14^+^ cells in both groups of donors [4]. The expression of this receptor was slightly lower on adherent monocytes of TST(+) than TST(-) volunteers. However, more homogenous monocyte fractions containing about 95% CD14^+^ cells isolated from TST(+) and TST(-) donors using immunomagnetic method with microparticles conjugated with anti- human CD14 monoclonal antibody (mAb) showed a similar expression of mCD14. A thorough analysis of the results revealed the significance of the method of isolating monocyte blood fractions in the evaluation of properties of tested cells. This suggestion is supported by the recently observed differences in the properties of dendritic cells derived from peripheral blood monocytes, that were isolated with different immunomagnetic methods and investigated by scanning electron microscopy [51] The height of dendritic cells derived from peripheral blood monocytes incubated before separation with anti-human CD14 mAb coated magnetic beads (positive separation) with the following differentiation in the presence of human granulocyte-macrophage colony stimulating factor, was significantly lower in comparison to monocytes non-incubated with anti-CD14 mAb (negative separation).

## Conclusions

In summary, our results suggest that CD14(-159C/T) polymorphic variants may play a role in controlling the level of response of the immune system regulating the development of DTH to PPD in individuals subjected to BCG immunization. Further studies on identifying the host genes responsible for TB resistance should provide new insights into the complex antimycobacterial immunity and lead to better understanding of the pathogenesis of TB and development of novel prophylactic or treatment strategies.
